# INPP5E Preserves Genomic Stability through Regulation of Mitosis

**DOI:** 10.1128/MCB.00500-16

**Published:** 2017-03-01

**Authors:** Elizabeth A. Sierra Potchanant, Donna Cerabona, Zahi Abdul Sater, Ying He, Zejin Sun, Jeff Gehlhausen, Grzegorz Nalepa

**Affiliations:** a Department of Pediatrics, Herman B Wells Center for Pediatric Research, Indiana University School of Medicine, Indianapolis, Indiana, USA; b Department of Biochemistry and Molecular Biology, Indiana University School of Medicine, Indianapolis, Indiana, USA; c Department of Medical and Molecular Genetics, Indiana University School of Medicine, Indianapolis, Indiana, USA; d Division of Pediatric Hematology-Oncology, Riley Hospital for Children, Indianapolis, Indiana, USA

**Keywords:** INPP5E, aneuploidy, cell cycle, centrosomes, mitosis, spindle assembly checkpoint

## Abstract

The partially understood phosphoinositide signaling cascade regulates multiple aspects of cellular metabolism. Previous studies revealed that INPP5E, the inositol polyphosphate-5-phosphatase that is mutated in the developmental disorders Joubert and MORM syndromes, is essential for the function of the primary cilium and maintenance of phosphoinositide balance in nondividing cells. Here, we report that INPP5E further contributes to cellular homeostasis by regulating cell division. We found that silencing or genetic knockout of *INPP5E* in human and murine cells impairs the spindle assembly checkpoint, centrosome and spindle function, and maintenance of chromosomal integrity. Consistent with a cell cycle regulatory role, we found that INPP5E expression is cell cycle dependent, peaking at mitotic entry. INPP5E localizes to centrosomes, chromosomes, and kinetochores in early mitosis and shuttles to the midzone spindle at mitotic exit. Our findings identify the previously unknown, essential role of INPP5E in mitosis and prevention of aneuploidy, providing a new perspective on the function of this phosphoinositide phosphatase in health and development.

## INTRODUCTION

Cellular safeguards against genomic instability include mitotic checkpoints that ensure faithful chromosome transmission across cell divisions. Aneuploidy due to error-prone mitosis promotes mistimed proliferation and cancer through a multifactorial impact on cellular metabolism (1). The spindle assembly checkpoint (SAC) prevents erratic chromosome segregation and aneuploidy by arresting the dividing cell in prometaphase until the dynamic kinetochore surveillance signaling network concludes that all kinetochores have achieved correct amphitelic attachment to mitotic spindle microtubules (2, 3). Satisfaction of the SAC activates the APC/C^CDC20^ ubiquitin ligase, which simultaneously decreases cyclin-dependent kinase activity by targeting cyclin B1 for proteolysis and uncouples sister chromatids by degrading the separase inhibitor securin, thus allowing the commencement of chromosome segregation.

**FIG 1:**
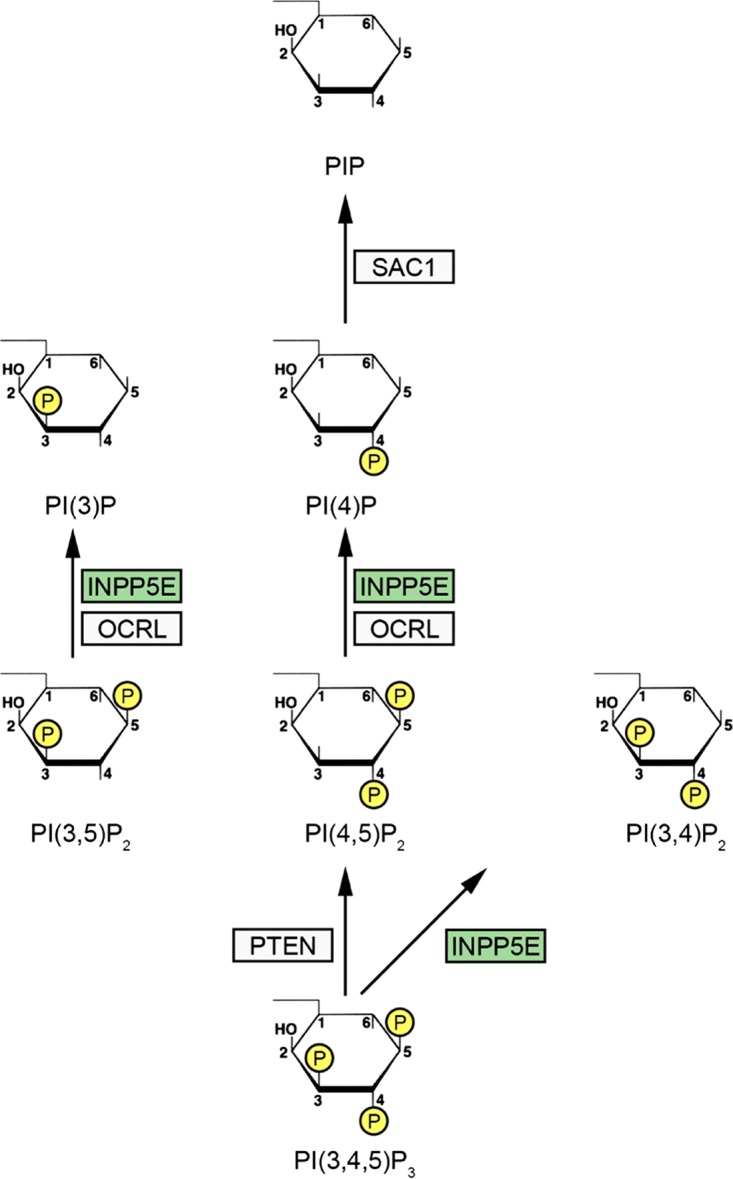
Phosphoinositide phosphatases that control mitosis. The complex network of phosphoinositide phosphatases and kinases that together regulate cell cycle progression and prevent human disease has been reviewed in detail elsewhere (see the text for references). Three phosphoinositide phosphatases (PTEN, INPP5E, and SAC1) are shown here in the context of the simplified phosphoinositide (PIP) signaling network, showing relevant primary phosphatase substrates. PTEN is an established tumor suppressor that controls chromosome segregation and negatively controls the mitogen-activated protein kinase (MAPK) signaling network. Inherited *PTEN* mutations occur in a variety of cancer predisposition/central nervous system (CNS) malformation syndromes with partially overlapping clinical phenotypes, including Cowden syndrome and Bannayan-Riley-Ruvalcaba syndrome. Congenital *OCRL* mutations are found in Lowe syndrome associated with ocular abnormalities, mental retardation, and renal dysfunction. The OCRL phosphatase performs multiple cellular functions, including control of mitotic exit by processing midbody-associated PIPs to locally reorganize the midbody cytoskeleton at abscission. Germ line *INPP5E* mutations contribute to Joubert/MORM ciliopathy syndromes in humans and cause severe perinatal lethality in mice, while acquired mutations within *INPP5E* (green) occur in a variety of cancers. The SAC1 phosphatase controls mitotic spindle assembly and function, and disruption of SacI causes embryonic lethality in mice. While the mechanistic role of these phosphoinositide phosphatases in PIP metabolism and regulation of cellular homeostasis needs to be dissected in detailed in future studies, the clinical phenotypes of *INPP5E*-, *PTEN*-, and *OCRL*-deficient humans highlight the essential role of these cell cycle-regulating PIP phosphatases in preventing developmental malformations and cancers.

Complete disruption of the SAC rapidly induces a degree of aneuploidy incompatible with cellular survival. Partial impairment of SAC fidelity regulators causes developmental abnormalities and promotes cancer. Through a genome-wide screen for SAC phosphatases, we identified the inositol polyphosphate-5-phosphatase INPP5E as a candidate regulator of this checkpoint (4). Phosphorylation of SAC proteins is well known to control checkpoint activity, but less is known about how phosphorylation of nonprotein signal messengers contributes to error-free mitosis. Phosphoinositides (PIs) are membrane-bound phospholipids composed of a polar inositol ring connected to hydrophobic fatty acid chains of a glycerophospholipid via a phosphate group. Dynamic differential phosphorylation and dephosphorylation of the PI inositide ring's three hydroxyl groups (3, 4, and 5) by the network of phosphoinositide kinases and phosphatases generates seven phosphoinositide phosphate (PIP) isoforms that play distinct roles in cellular metabolism (Fig. 1) (5). INPP5E dephosphorylates the 5′-hydroxyl group of the inositol ring in PI(3,4,5)P_3_, PI(3,5)P_2_, and PI(4,5)P_2_, which are thought to act as localized protein recruiters to impact numerous processes, including cell division (6). Previous studies implicated strict control of the spatiotemporal distribution of phosphoinositides in mitosis, from the cell rounding that accompanies the onset of mitosis to mitotic spindle function and execution of cytokinesis (7). Since a disordered cell cycle promotes chromosomal instability and malignant transformation, it is not surprising that several phosphoinositide-processing enzymes are implicated in cancer. Loss of the inositol polyphosphate 4-phosphatase INPP4B stimulates tumorigenesis *in vivo* at least partially through hyperactivation of the phosphoinositide-regulated AKT-SGK3 signaling axis (8–10). Interestingly, overexpression of INPP4B may paradoxically promote tumorigenesis in acute myeloid leukemia independently of the INPP4B phosphatase activity through mechanisms that remain to be explained (11, 12). The inositol polyphosphate 4-phosphatase PTEN (phosphatase and tensin homolog) is an established tumor suppressor (reviewed in reference 13). However, the role of INPP5E and other phosphoinositide-5-phosphatases in tumorigenesis is less clear: both up- and downregulation of these enzymes have been reported in cancer (14, 15). Further, germ line *INPP5E* mutations occur in a fraction of patients with Joubert and MORM (mental retardation, obesity, retinal dystrophy, and micropenis) developmental syndromes (16–18), although the pathogenesis of these disorders is not fully understood from the mechanistic standpoint. Previous studies have demonstrated that INPP5E regulates ciliary function in nondividing cells, but the role of this phosphatase during cell division had not been examined in detail.

In this work, we demonstrate that INPP5E is essential for normal mitosis and the SAC and that loss of INPP5E promotes genomic instability. We show that INPP5E expression is cell cycle dependent and that INPP5E shuttles to the mitotic apparatus in dividing cells to impact centrosome and spindle function. These novel roles of INPP5E in cell division may be related to the roles of this phosphatase in development and cancer.

## RESULTS

### INPP5E is essential for the spindle assembly checkpoint.

**FIG 2:**
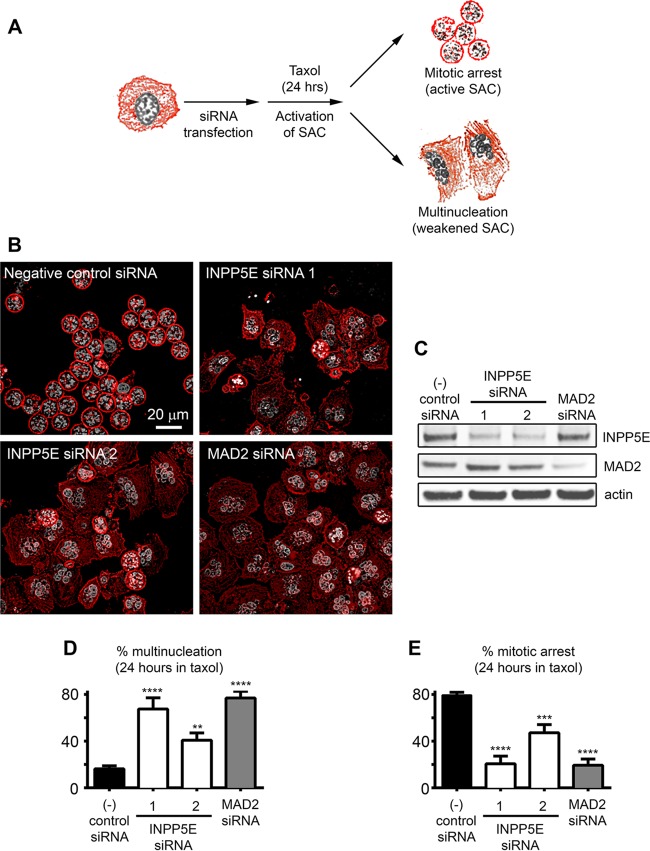
INPP5E regulates the spindle assembly checkpoint. (A) Assay schematic. Deficient SAC promotes multinucleation in paclitaxel-exposed cells. (B) Multinucleation due to impaired SAC in *INPP5E* and *MAD2* knockdown cells exposed to paclitaxel. Note prometaphase arrest (active SAC) in control cells (condensed chromosomes in round mitotic cells). (C) Target knockout confirmed by Western blotting. (D and E) Quantification of multinucleation and mitotic arrest, respectively. One-way analysis of variance (ANOVA) was used to calculate *P* values (*n* ≥ 4 counts/siRNA). **, *P* < 0.01; ***, *P* < 0.001; ****, *P* < 0.0001.

**FIG 3:**
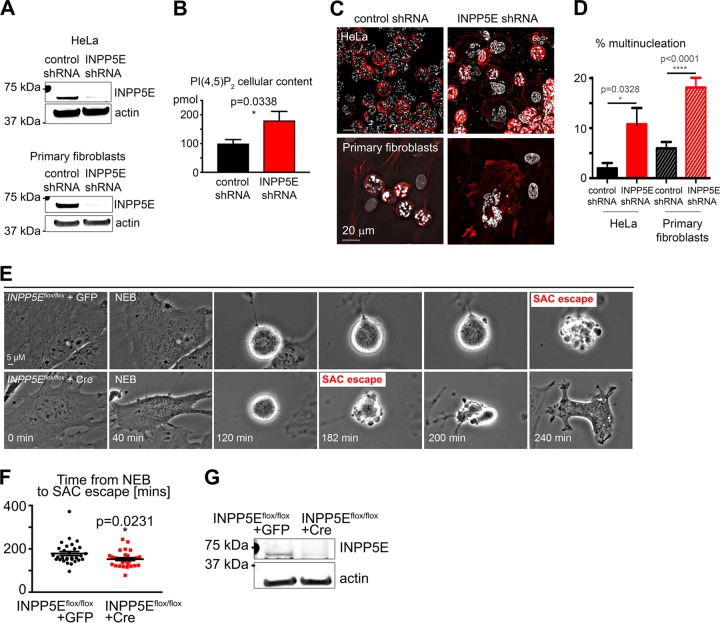
Stable *INPP5E* knockdown weakens the SAC in HeLa cells and primary human fibroblasts. (A) INPP5E levels in cell lines stably expressing the indicated shRNAs. (B) Accumulation of an INPP5E substrate, PI(4,5)P_2_, in *INPP5E* knockdown HeLa cells. (C) Representative images of the indicated cell lines treated with paclitaxel for 22 h. Note multinucleation reflecting a weakened SAC in INPP5E-deficient HeLa cells and fibroblasts. (D) Quantification of SAC assay results. (E) Representative time-lapse images of *Inpp5e^flox/flox^* MEFs transduced with negative-control GFP lentivirus (top panel) and GFP-Cre recombinase (bottom panel) following paclitaxel exposure. Note accelerated SAC escape in the *Inpp5e^flox/flox^* cell. (F) Quantification of the length of time between NEB and SAC escape. The *P* value was calculated with an unpaired *t* test. For both cell types, *n* = 30 (two pooled experiments). (G) Western blot of whole-cell lysates from *Inpp5e^flox/flox^* MEFs transduced with lentivirus encoding GFP control or GFP-fused Cre recombinase.

We identified INPP5E as a candidate SAC regulator in an unbiased genome-wide small interfering RNA (siRNA) screen aimed to identify phosphatases controlling mitosis (4). To test whether INPP5E is required for the SAC (Fig. 2A), we employed two independent *INPP5E* siRNAs validated by quantitative Western blotting (Fig. 2C). HeLa cells were transfected with the indicated siRNAs, and the SAC was activated with the microtubule-stabilizing drug paclitaxel (originally named taxol). Cells were then fixed and examined for SAC maintenance (Fig. 2B) in the quantitative multinucleation assay that we have previously described (4). While negative-control cells maintained checkpoint arrest, cells transfected with siRNA against the SAC regulator and tumor suppressor MAD2 (mitotic arrest deficient-like 2) (19) exhibited extensive multinucleation. Similarly, *INPP5E* knockdown promoted escape from the SAC (Fig. 2B to E). Stable short hairpin RNA (shRNA)-mediated *INPP5E* knockdown also impaired the SAC in human fibroblasts and HeLa cells (Fig. 3). INPP5E deficiency results in increased levels of its phosphoinositide substrates (17). To confirm that INPP5E phosphatase activity is depleted upon *INPP5E* knockdown, we confirmed that HeLa cells stably expressing *INPP5E* shRNA contain more total PI(4,5)P_2_ (an INPP5E phosphoinositide substrate) than control cells as determined by using a quantitative enzyme-linked immunosorbent assay (ELISA) (Fig. 3C). To verify that impairment of the SAC was due to depletion of INPP5E, we quantified the SAC efficiency upon Cre-mediated depletion of Inpp5e in *Inpp5e*^*flox/flox*^ mouse embryonic fibroblasts (MEFs) (17). Live imaging revealed shortened paclitaxel-induced SAC arrest in *Inpp5e* knockout MEFs (Fig. 3E and F). Western blotting confirmed Inpp5e knockout upon Cre expression in *Inpp5e*^*flox/flox*^ MEFs (Fig. 3G). We concluded that *INPP5E* knockdown impairs SAC function.

### The INPP5E substrate PI(4,5)P_2_ promotes SAC escape.

**FIG 4:**
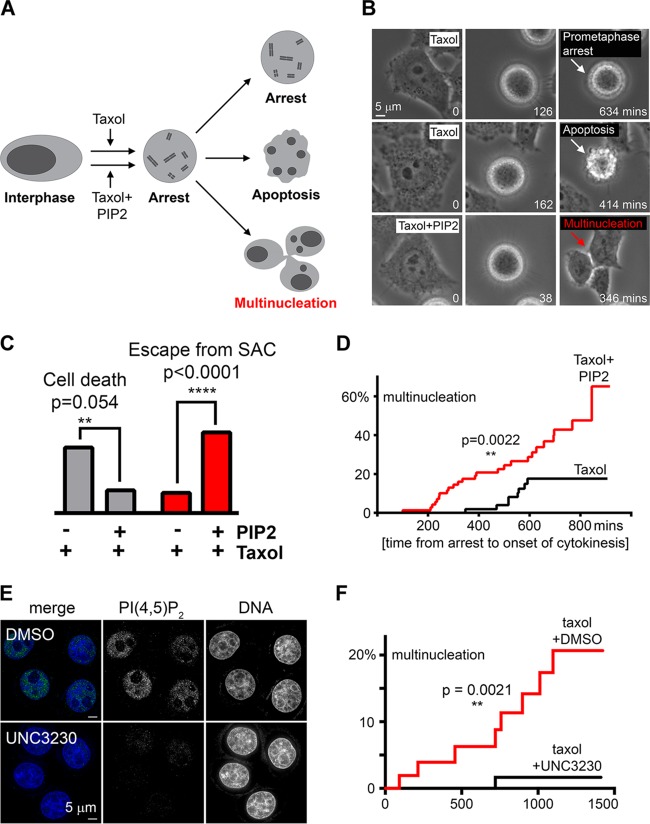
Excess PI(4,5)P_2_ impairs the SAC. (A) Assay schematic. Prolonged SAC arrest triggers cell death unless checkpoint escape occurs. (B) Representative time-lapse images of cells treated with paclitaxel alone versus paclitaxel plus PI(4,5)P_2_. (C) Cells treated with paclitaxel plus PI(4,5)P_2_ are less likely to die and more likely to escape SAC upon prolonged arrest within 24 h of mitotic entry than cells treated with paclitaxel alone (*n* = 100 arrested cells tracked via time-lapse imaging per condition; *P* values were calculated with Fisher's exact test). Percentages of categorical values are shown. (D) Cumulative incidence of SAC escape in cells treated with paclitaxel plus DMSO versus paclitaxel plus PI(4,5)P_2_. The *P* value for risk of SAC escape was calculated with the log rank Mantel-Cox test. (E) Representative images of HeLa cells stably expressing shRNA against *INPP5E* stained with a PI(4,5)P_2_-specific antibody after a 24-hour exposure to DMSO (top) or the PIP5K1C/PIP4K2C inhibitor UNC3230 (100 nM) (bottom). Note decreased nuclear PI(4,5)P_2_ in UNC3230-treated cells. (F) Cumulative incidence of SAC escape in stable *INPP5E* knockdown HeLa cells treated with paclitaxel plus DMSO versus paclitaxel plus UNC3230. The *P* value for risk of SAC escape was calculated with the log rank Mantel-Cox test. For DMSO- and UNC3230-treated cells, *n* = 53 and 60, respectively.

Since *INPP5E* knockdown promotes accumulation of PI(4,5)P_2_ (Fig. 3B), we asked if treatment with excess PI(4,5)P_2_ affected the SAC (Fig. 4A). Exposure to high-dose paclitaxel alone induced prolonged SAC arrest leading to cell death (20), but excess PI(4,5)P_2_ promoted escape from paclitaxel-induced death, premature mitotic exit, and multinucleation (Fig. 4B and C). Furthermore, PI(4,5)P_2_ reduced the duration of paclitaxel-induced checkpoint arrest (Fig. 4D). Thus, elevated levels of PI(4,5)P_2_ impair the SAC, suggesting one potential mechanism of SAC failure resulting from INPP5E deficiency (Fig. 2). To determine whether reducing PI(4,5)P_2_ rescues the SAC impairment in INPP5E-deficient cells, we sought to inhibit cellular synthesis of PI(4,5)P_2_. Recent studies identified a selective small-molecule inhibitor of phosphatidylinositol 4-phosphate 5-kinase 1 gamma (PIP5K1C) and phosphatidylinositol-5-phosphate 4-kinase 2 gamma (PIP4K2C), kinases that convert PI(4)P and PI(5)P, respectively, to PI(4,5)P_2_ (21, 22). The authors demonstrated that this inhibitor, UNC3230, reduced PI(4,5)P_2_ staining in neurons cultured *ex vivo* (22). We found that treatment with UNC3230 reduced PIP(4,5)P_2_ staining in HeLa *INPP5E* knockdown cells (Fig. 4E). Live imaging revealed that pretreatment with UNC3230 significantly decreased the number of *INPP5E* knockdown cells that escaped SAC arrest (Fig. 4F). Overall, these findings suggests that disequilibrium of PI(4,5)P_2_ contributes to an impaired SAC in INPP5E-deficient cells.

### INPP5E is required for normal mitotic progression.

**FIG 5:**
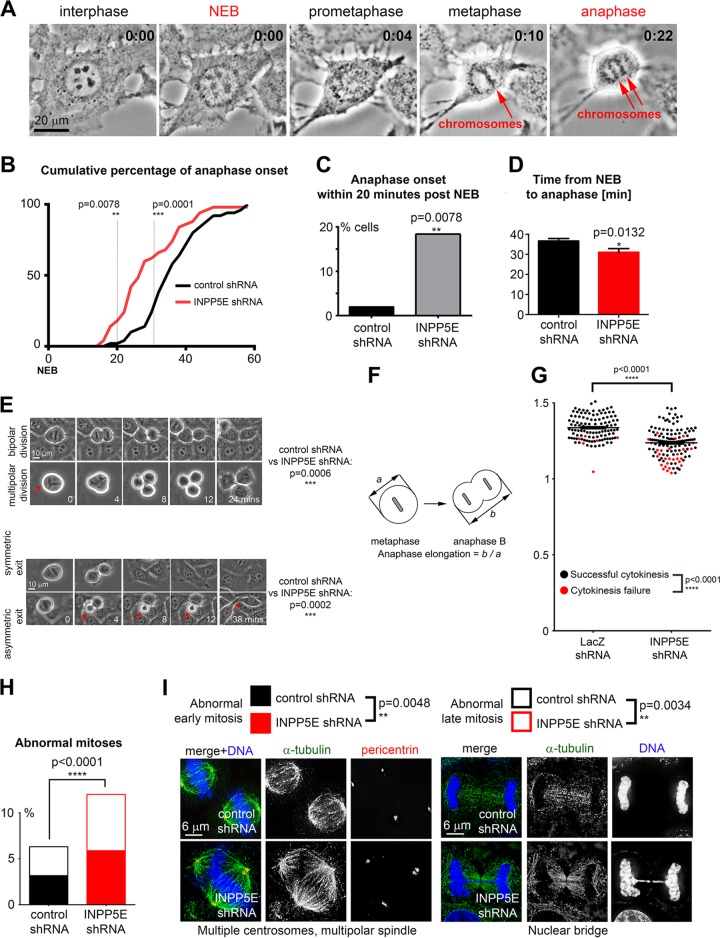
INPP5E controls unperturbed mitosis. (A) Representative time-lapse imaging of a control cell progressing through mitosis. (B)Cumulative percentage of anaphase onset after NEB. *P* values were calculated with Fisher's exact test. (C) Fraction of anaphase cells 20 min after NEB (*P* = 0.0078 by Fisher's exact test; percentages of categorical values are shown). (D) Silencing *INPP5E* accelerates progression from NEB to anaphase. The *P* value was calculated with an unpaired *t* test (*n* = 50 control cells and 51 *INPP5E* knockdown cells). (E) Knockdown of *INPP5E* increases frequency of multipolar divisions and asymmetric mitotic exit in HeLa cells examined via time-lapse video microscopy compared to cells expressing nontargeting shRNA. *P* values were calculated with Fisher's exact test for multipolar divisions and with the chi-square test with Yates' correction for asymmetric mitotic exit. At least 500 dividing cells were quantified per genotype and condition. (F) Anaphase spindle elongation assay design. Cells were monitored through mitosis via time-lapse imaging, and the ratio of anaphase B length to metaphase length was determined for each cell to quantify the efficiency of anaphase spindle elongation similarly to previously described assays (63, 64). (G) Knockdown of *INPP5E* decreases anaphase spindle elongation in HeLa cells (dot plot, *P* < 0.0001 in two-tailed *t* test, *n* = 117 control cells and 132 *INPP5E* shRNA cells) and increases the frequency of cytokinesis failure (red dots, *P* < 0.0001 in Fisher's exact test) in HeLa *INPP5E* knockdown cells compared to cells transfected with negative-control siRNA. Each dot represents a single cell. (H) INPP5E-deficient cells undergo abnormal mitosis more frequently than control cells. (I) Examples of abnormal mitotic figures in *INPP5E* knockdown cells compared to controls. *P* values for early mitosis (prophase through metaphase) and late mitosis (anaphase through cytokinesis) are shown.

**FIG 6:**
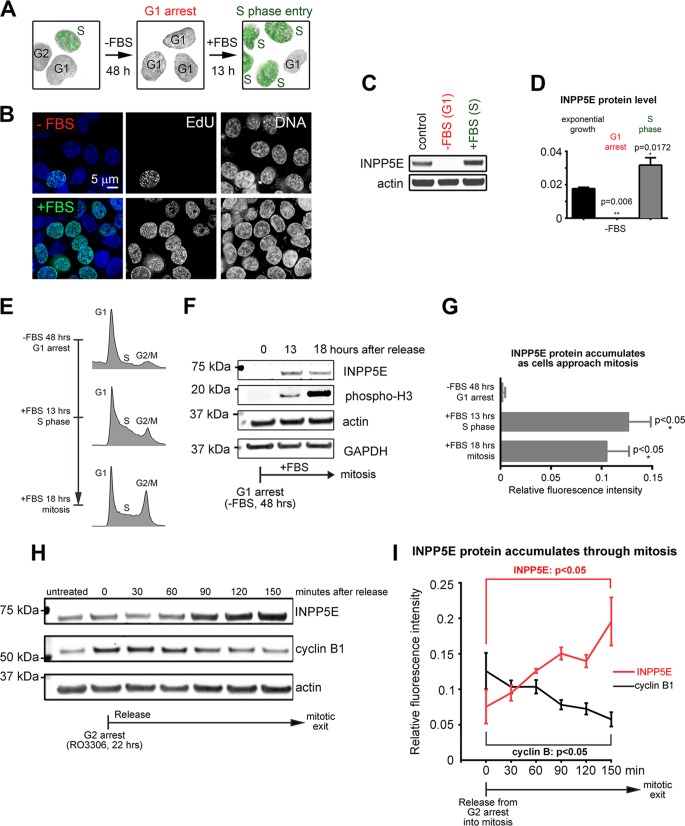
INPP5E protein expression is cell cycle dependent. (A and B) Serum starvation-induced G_1_ arrest in HCT cells. (C) Decreased endogenous INPP5E in HCT cells arrested in G_1_ via serum starvation. (D) Quantification of infrared Western blotting results shown in panel C. *P* values were calculated via ANOVA with Sidak's multiple-comparison test (3 replicates). (E) Representative cell cycle flow cytometry profiles of cells at the indicated time points after release from starvation-induced G_1_ arrest. (F) INPP5E accumulates as cells progress through the cell cycle. (G) Quantification of INPP5E protein levels at the indicated time points. *P* values for INPP5E were determined using unpaired *t* tests (*n* = 3 replicates). (H) Western blots of HeLa cell lysates released from RO3306-induced G_2_ arrest at the indicated time points. (I) Quantification of INPP5E and cyclin B1 protein (normalized to actin, *n* = 3 replicates) at the indicated time points. *P* values were calculated by one-way ANOVA.

Since INPP5E regulated the paclitaxel-induced SAC response, we asked whether INPP5E deficiency impaired mitosis in unperturbed cells. We used time-lapse imaging to quantify time from nuclear envelope breakdown (NEB) to anaphase onset (Fig. 5A). INPP5E-deficient cells progressed from NEB to anaphase at an increased speed (Fig. 5B to D). We noticed that silencing *INPP5E* promoted multipolar divisions and asymmetric mitotic exit, with one of the daughter cells requiring more time to complete the exit from mitosis (Fig. 5E). We found that INPP5E is required for anaphase spindle elongation and completion of cytokinesis (Fig. 5F and G). We examined INPP5E-deficient cells through deconvolution microscopy to evaluate these structural mitotic defects in more detail. In agreement with our live imaging data (Fig. 6A to G), INPP5E deficiency promoted early mitotic defects (multipolar spindles and supernumerary centrosomes) (Fig. 5H) as well as late mitotic abnormalities such as abnormal anaphases with nuclear bridges and cytokinesis failure (Fig. 5I).

### INPP5E expression and subcellular localization are cell cycle dependent.

Given the newly found role of INPP5E in cell division, we next examined whether expression of INPP5E was cell cycle dependent. Consistent with the novel role for INPP5E in mitosis, the INPP5E level is low in serum-starved, G_1_-arrested cells (Fig. 6A to D) but rises as cells progress through S and G_2_ toward mitotic entry (Fig. 6E to G) and peaks in mitosis (Fig. 6H and I).

**FIG 7:**
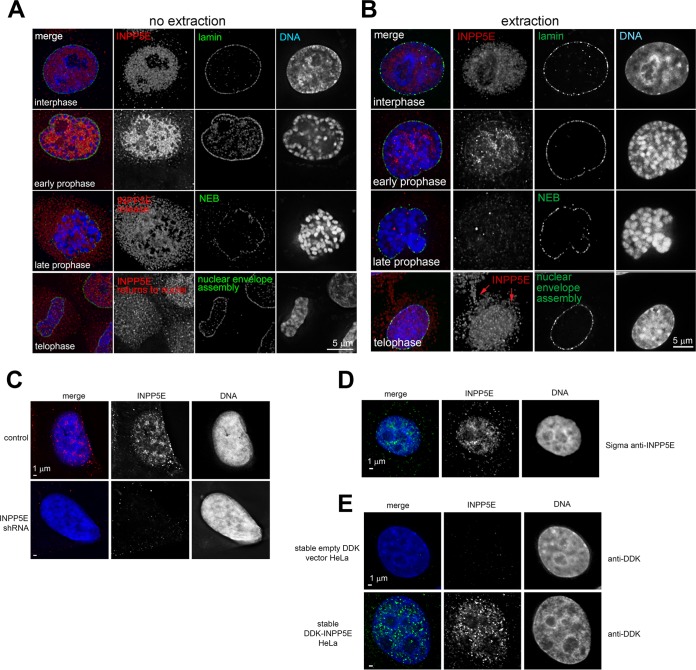
Nuclear localization of INPP5E in interphase and mitosis. (A and B) Representative images of soluble (A) and insoluble (B) fractions of endogenous INPP5E in interphase and mitosis. Note that detergent extraction removes most of the soluble endogenous INPP5E after nuclear envelope breakdown in late prophase. Insoluble INPP5E returns to nucleus upon nuclear envelope reassembly in telophase. Images are representative of a total of least 30 cells across three separate experiments. (C) Decreased nuclear INPP5E immunofluorescence signal upon shRNA-mediated *INPP5E* knockdown. (D) Validation of endogenous INPP5E nuclear localization via another anti-INPP5E primary antibody. (E) Stably overexpressed DDK-INPP5E localizes to the nucleus.

**FIG 8:**
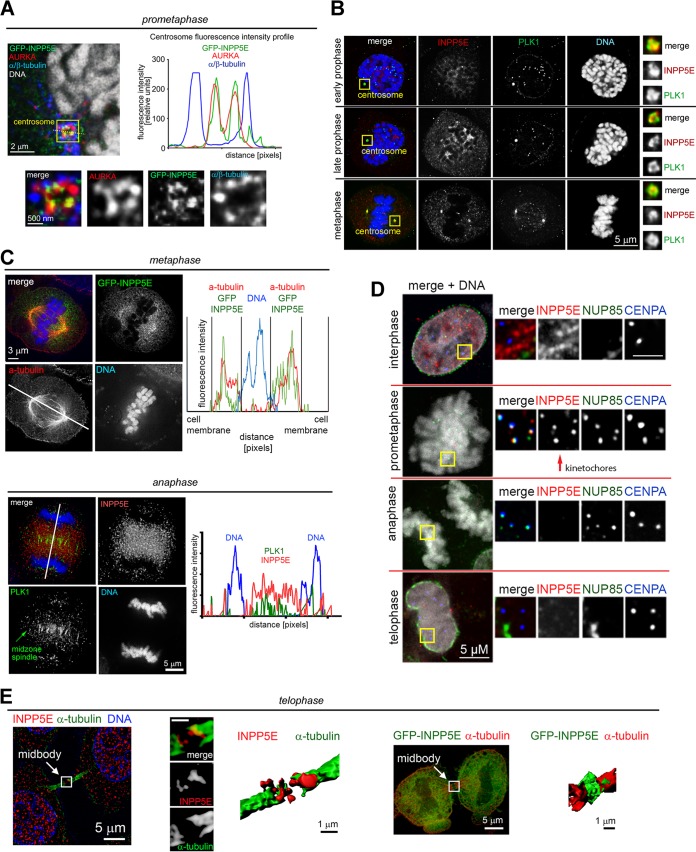
INPP5E localizes to mitotic structures. (A) The insoluble fraction of endogenous INPP5E associates with mitotic centrosomes as demonstrated by colocalization with Aurora kinase A proximal to centrosome-microtubule attachment sites (immunofluorescence line intensity profiles shown as insert). (B) Coimmunofluorescence with Polo-like kinase PLK1 validates localization of endogenous INPP5E to mitotic centrosomes. (C) A fraction of extraction-resistant endogenous INPP5E binds prometaphase kinetochores, as demonstrated by coimmunofluorescence with the kinetochore markers NUP85 and CENPA. Note that INPP5E kinetochore localization is cell cycle dependent and decreases after anaphase entry. (D) Endogenous INPP5E is enriched around the metaphase and anaphase spindle as demonstrated by coimmunofluorescence with alpha-tubulin and PLK1, respectively. Immunofluorescence line intensity profiles are shown. (E). INPP5E shuttles to the midbody during telophase. Alpha-tubulin was used as a midbody marker to demonstrate localization of endogenous INPP5E (left) and GFP-INPP5E (right). Three-dimensional midbody models were generated in Imaris. HeLa cells were used for all images shown. Images are representative of at least 3 cells per cell cycle phase from two experiments.

We examined subcellular INPP5E localization throughout the cell cycle via deconvolution and superresolution structured illumination microscopy (SR-SIM). Consistent with known ciliary localization of INPP5E (17), we found INPP5E at interphase centrioles (data not shown). Overexpressed INPP5E localized to selected biological membranes (data not shown). A fraction of INPP5E was present inside the nucleus (Fig. 7). In agreement with our biochemistry data (Fig. 6), nuclear INPP5E immunofluorescence increased in prophase (Fig. 7A). Upon nuclear envelope breakdown, INPP5E diffused throughout the cell, with a fraction of INPP5E accumulating around chromosomes (data not shown). Detergent extraction prior to fixation removed diffuse INPP5E from mitotic cells (Fig. 7B) and revealed that a fraction of INPP5E colocalizes at mitotic centrosomes with Aurora kinase A (AURKA), a known INPP5E-interacting protein (23) (Fig. 8A), and Polo-like kinase 1 (PLK1) (24) (Fig. 8B). More-detailed analysis of cross sections through prometaphase centrosomes showed that INPP5E colocalizes with AURKA at the pericentriolar material (PCM) proximal to spindle microtubule attachment sites (Fig. 8A). A fraction of insoluble INPP5E colocalized with kinetochore markers NUP85 and centromere protein A (CENPA) during prometaphase (Fig. 8D). In metaphase, the spindle was surrounded by diffuse INPP5E, which shuttled to the midzone in anaphase (Fig. 8C, top panel). By anaphase onset, the extraction-resistant fraction of INPP5E dissociated from kinetochores (Fig. 8C, bottom panel). INPP5E was observed at the midbody during telophase (Fig. 8E), with a large fraction returning to the nuclei upon nuclear envelope reassembly (Fig. 7 and 8).

**FIG 9:**
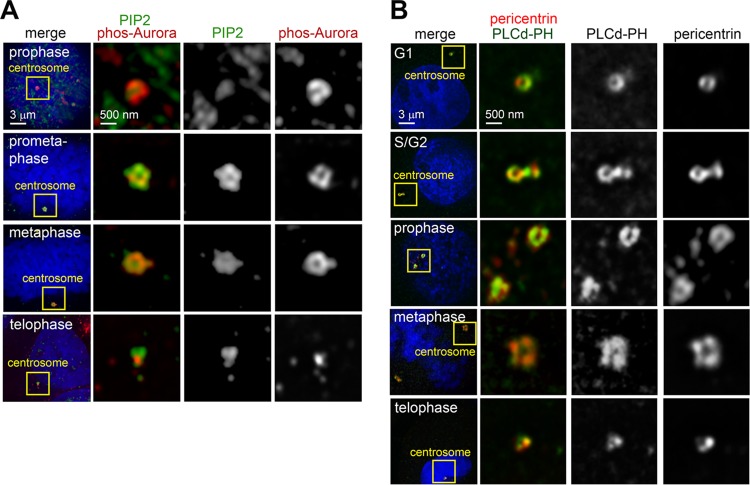
Phosphoinositides localize to mitotic centrosomes. (A) HeLa cells stained with a PI(4,5)P_2_-specific antibody and anti-phospho-Aurora to mark mitotic centrosomes. (B) Representative images of HeLa cells transiently expressing an RFP-fused construct of the PI(4,5)P_2_- and PI(3,4,5)P_3_-binding domain of phospholipase C (PLCδ-PH). Cells were costained with antipericentrin to mark centrosomes.

We wondered whether the INPP5E phosphoinositide substrates were also present at mitotic centrosomes. Immunofluorescence using a PI(4,5)P_2_-specific antibody demonstrated that PI(4,5)P_2_ was present at centrosomes throughout mitosis (Fig. 9A). We validated this finding by imaging HeLa cells transfected with a red fluorescent protein (RFP)-fused phospholipase C δ (PLCδ)-pleckstrin homology (PH) domain, which binds PI(3,4,5)P_3_ and PI(4,5)P_2_ (25) (Fig. 9B). A fraction of RFP–PLCδ-PH colocalized with the centrosomal marker pericentrin (PCNT) during cell division (Fig. 9B). These observations are consistent with a role for INPP5E and its substrates at the centrosome.

### INPP5E regulates the function of the chromosome-segregating apparatus.

**FIG 10:**
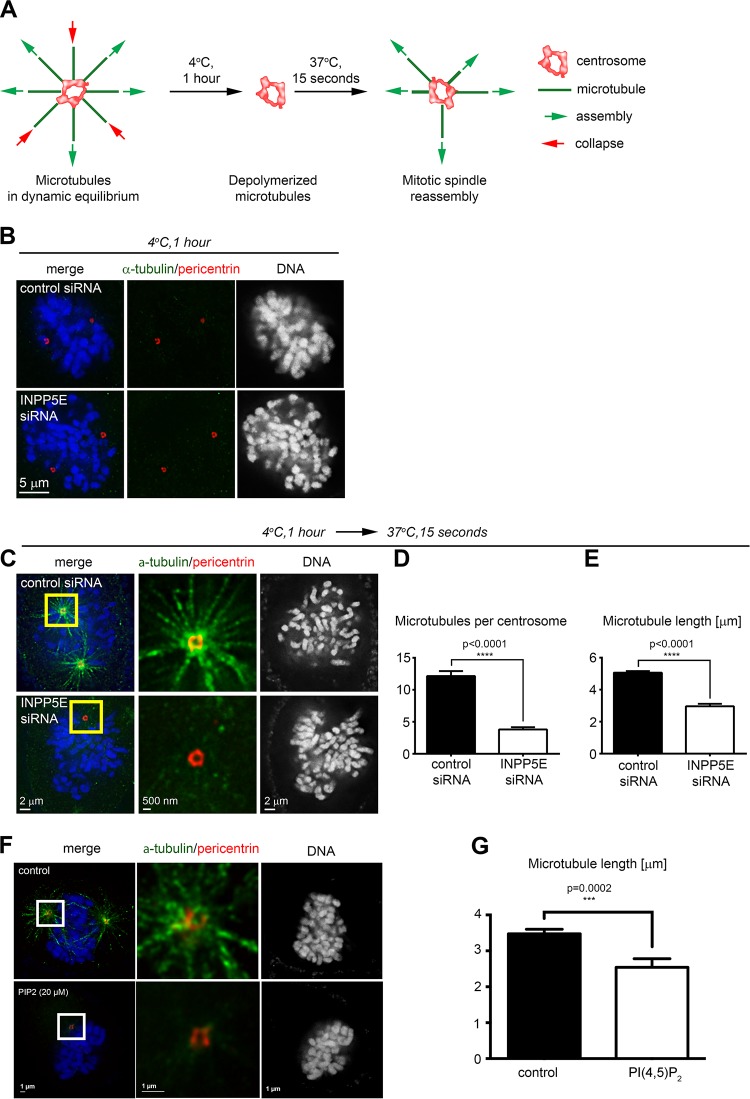
INPP5E regulates nucleation of spindle microtubules at mitotic centrosomes. (A) Assay schematic. (B) Cold spindle destabilization in control and *INPP5E* knockdown prometaphase cells. (C) Impaired microtubule spindle reassembly in a representative *INPP5E* knockdown cell compared to a control. (D and E) Quantification of the number of microtubules per centrosome (D) and the microtubule length (E). *P* values were calculated by two-tailed *t* tests (*n* = 14 centrosomes/170 microtubules for controls and 20 centrosomes/76 microtubules for *INPP5E* knockdown cells). (F) Representative image of prometaphase microtubule repolymerization in cold-treated cells following treatment with carrier only (top panel) or 20 μM PI(4,5)P_2_ (bottom panel). (G) Quantification of microtubule length. the *P* value was calculated with an unpaired *t* test. For control cells, *n* = 175; for PI(4,5)P_2_-treated cells, *n* = 76.

Since INPP5E localizes to the PCM, the organized proteinaceous network (26) responsible for spindle assembly (27, 28), we asked whether *INPP5E* knockdown affected the centrosome's ability to nucleate microtubules in modified cold spindle destabilization assays (29) (Fig. 10A). Live HeLa cells were cold treated to disrupt microtubules (Fig. 10B), allowed a brief recovery in warm medium, fixed, immunostained, and imaged to assess microtubule repolymerization. *INPP5E* knockdown impaired microtubule nucleation from mitotic centrosomes (Fig. 10C to E). Quantification confirmed that *INPP5E* knockdown reduced both the number (Fig. 10D) and length (Fig. 10E) of spindle microtubules. We validated this phenotype in CRISPR (clustered regularly interspaced short palindromic repeat)/Cas-edited *INPP5E* knockout cells (data not shown). HeLa cells treated with PI(4,5)P_2_ exhibited similar microtubule nucleation defects (Fig. 10F and G), suggesting that the abnormal mitotic progression in INPP5E-deficient cells may be at least partially due to a disrupted phosphoinositide balance. Quantitative live imaging demonstrated impaired anaphase spindle elongation in *INPP5E* knockdown HeLa cells (Fig. 6F and G), providing further evidence of spindle malfunction resulting from INPP5E deficiency.

### Knockdown of *INPP5E* promotes genomic instability.

**FIG 11:**
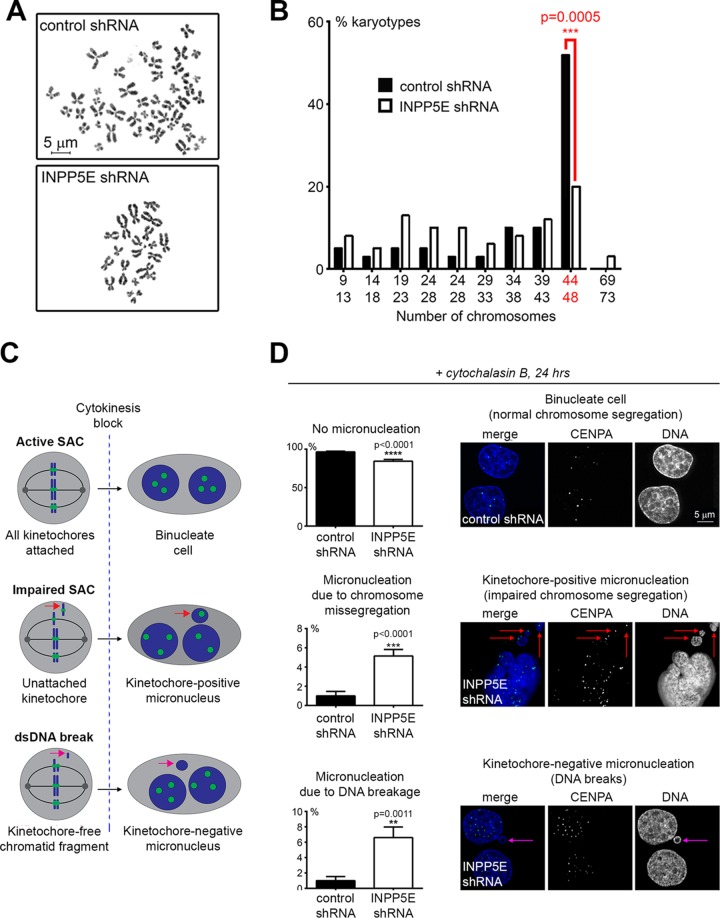
Loss of INPP5E causes genomic instability. (A and B) Chromosome instability in INPP5E-deficient cells. Representative metaphase chromosome spreads prepared from stable control and *INPP5E* shRNA-expressing primary human early-passage fibroblasts are shown. For quantification of abnormal karyotypes (B), the *P* value was calculated with the two-tailed Fisher's exact test (*n* = 50/genotype). (C) Design of cytochalasin B micronucleation assay. (D) Micronucleation assay quantification. *P* values were determined via the two-tailed Fisher's exact test (*n* = 12 counts/phenotype/genotype). Representative micronuclei are shown.

Aneuploidy is a potential consequence of impaired mitosis; therefore, we sought to examine the effect of *INPP5E* knockdown on genomic stability. To directly assess the effect of knockdown of *INPP5E* on ploidy, we examined metaphase chromosome spreads in the stable fibroblast lines. INPP5E-deficient cells frequently exhibited an abnormal chromosome number (Fig. 11A) associated with a significant increase in the percentage of aneuploid cells (Fig. 11B).

To determine whether SAC impairment contributed to aneuploidy in INPP5E-deficient cells, we performed a cytochalasin B micronucleus assay as previously described (30). The control and *INPP5E* knockdown lines were treated with the cytokinesis-blocking drug cytochalasin B and subsequently assessed for micronucleus formation via fluorescence microscopy. To distinguish between micronuclei arising from whole misseggregated chromosomes (i.e., SAC failure) and micronuclei comprised of chromatid fragments resulting from unrepaired DNA double-strand breaks, the cells were immunostained for CENPA as a marker of kinetochores. Micronuclei were quantified as kinetochore positive or kinetochore negative (Fig. 11C). *INPP5E* knockdown resulted in a significant increase in the percentage of cells with both kinetochore-positive and kinetochore-negative micronuclei (Fig. 11D). These observations suggest that knockdown of *INPP5E* causes aneuploidy through both impairment of the SAC and unrepaired chromosomal breakage.

## DISCUSSION

Error-free mitosis prevents congenital abnormalities and cancer (1). Mitotic failure during embryonic growth leads to spontaneous abortion as well as a variety of developmental syndromes (31). After birth, chromosomal instability promotes carcinogenesis (32). Mitotic checkpoints that ensure faithful chromosome transmission across cell divisions provide safeguards against genomic instability. The SAC is one such key genomic guardian, and our genome-wide RNA interference (RNAi) screen identified INPP5E as a candidate regulator of the SAC (33).

We found that INPP5E functions outside interphase as a regulator of paclitaxel-triggered SAC (Fig. 2) and multiple stages of mitosis (Fig. 5). We confirmed the mitotic phenotype of impaired INPP5E expression with multiple distinct *INPP5E* siRNAs in HeLa cells, *INPP5E*-targeting shRNAs in HeLa cells and human fibroblasts, and *Inpp5e* knockout MEFs (Fig. 2 and 3 and data not shown). Further, we confirmed that *INPP5E* siRNAs did not impact *MAD2* (data not shown), whose transcript is sensitive to nonspecific siRNA silencing (34). INPP5E overexpression caused cellular toxicity, as described before (references 6 and 35 and data not shown). Consistent with its role in cell division, INPP5E accumulates as cells progress through interphase toward mitosis (Fig. 6). INPP5E maintains the function of centrosomes and the spindle during cell division (Fig. 8 and 10), which suggests an explanation for abnormal mitosis resulting from INPP5E deficiency. Interestingly, a recent study revealed an interaction between INPP5E and AURKA, a key mitotic kinase that controls centrosome maturation, mitotic progression, and ciliary stability (23). In support of these findings, we found that INPP5E colocalizes with PLK1 and AURKA at the PCM in early mitosis (Fig. 8).

**FIG 12:**
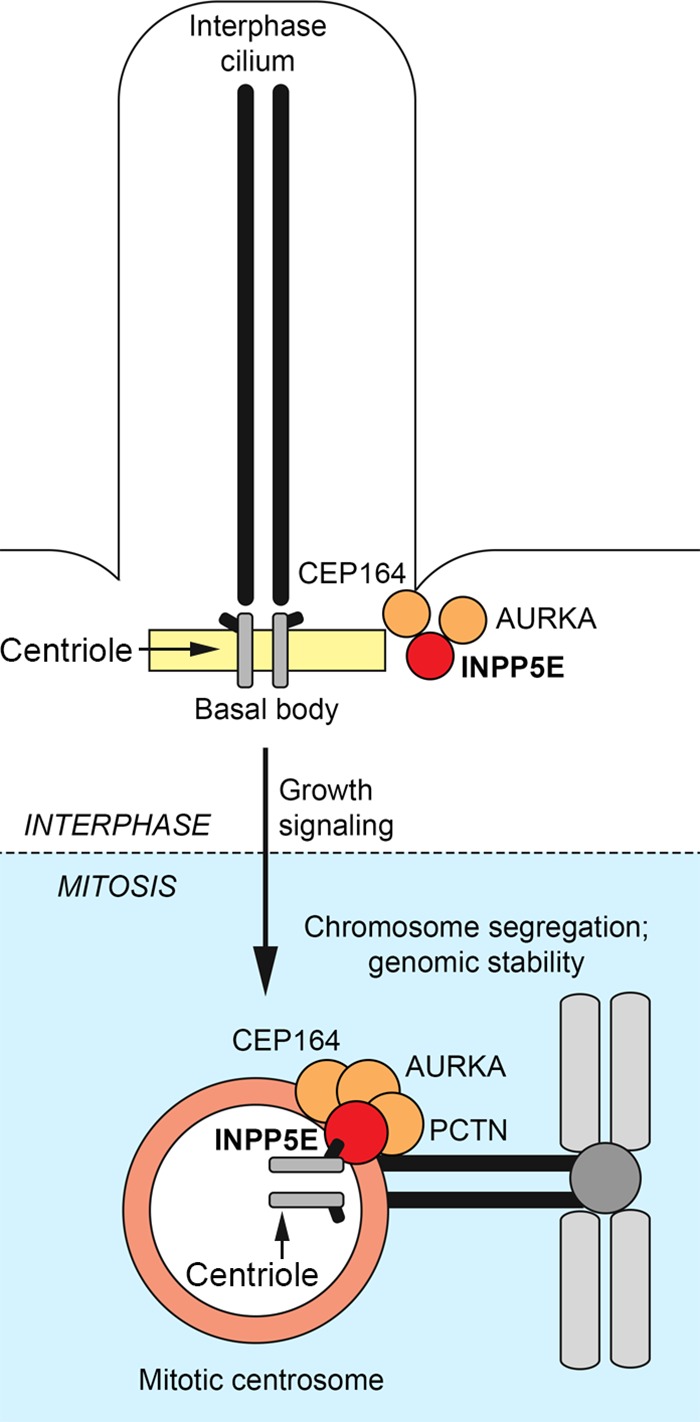
INPP5E controls cellular homeostasis by regulating cilia and centrosomes throughout the cell cycle. INPP5E regulates ciliary stability in interphase and controls mitotic apparatus during cell division. See the text for discussion.

Centrosomes and the basal body of cilia are assembled on the same core molecular framework that undergoes extensive structural and functional reorganization in response to cell cycle cues (36–40). Therefore, it is not surprising that the cilium-associated INPP5E regulates function of the primary cilia in interphase (17, 18) and centrosome activity in mitosis (Fig. 12). Since INPP5E is enriched at kinetochores, the spindle, the midbody, and centrosomes (Fig. 8 and 9), future studies will likely identify additional mitotic roles for INPP5E. Future work will also determine whether abnormal spindle function and orientation (Fig. 6 and 11) contribute to dysmorphogenesis (41, 42) in human congenital INPP5E deficiency syndromes, similar to the spindle malfunction mechanisms proposed in other ciliopathies (41, 42).

Phosphoinositides and their regulatory phosphatases contribute to multiple cellular processes, from cytoskeletal rearrangements to cell motility/adhesion, membrane trafficking, signal transduction (including Ca^2+^/AKT signaling), and gene expression (43–46). Loss of INPP5E promotes accumulation of PI(4,5)P_2_ (23). We found that PI(4,5)P_2_ localizes to mitotic centrosomes and impairs the SAC (Fig. 4 and 10), offering one potential explanation for the dysfunctional SAC in INPP5E-deficient cells, although it remains to be investigated whether the mitotic function of INPP5E is entirely phosphoinositide dependent and whether other INPP5E-regulated phosphoinositides contribute to mitotic progression. Interestingly, *INPP5E*-truncating mutations that do not affect phosphatase activity but disrupt ciliary localization by removing the C-terminal CAAX domain cause MORM syndrome, indicating that phosphoinositide-directed enzymatic activity of INPP5E is not sufficient to prevent disease if the phosphatase's subcellular targeting is disrupted (17).

Consistent with a role in mitosis, we found that INPP5E prevents aneuploidy. *INPP5E* knockdown caused chromosomal instability in primary human fibroblasts (Fig. 11A and B). Micronucleus assays revealed increased frequencies of both mitotic errors and unrepaired double-strand DNA breaks in INPP5E-deficient cells (Fig. 11C and D). Germ line mutations of two critical DNA damage response (DDR)-orchestrating phosphatidylinositol kinase-related kinase (PIKK) family kinases, ATM and ATR, cause progressive cerebellar dysfunction in ataxia-telangiectasia (47) or microcephaly with dwarfism in Seckel syndrome type I, respectively (48) in, addition to DNA damage hypersensitivity. INPP5E is phosphorylated at ATM/ATR recognition sites following exposure to ionizing radiation (49). Thus, it will be interesting to determine whether INPP5E contributes to the DDR as well.

**FIG 13:**
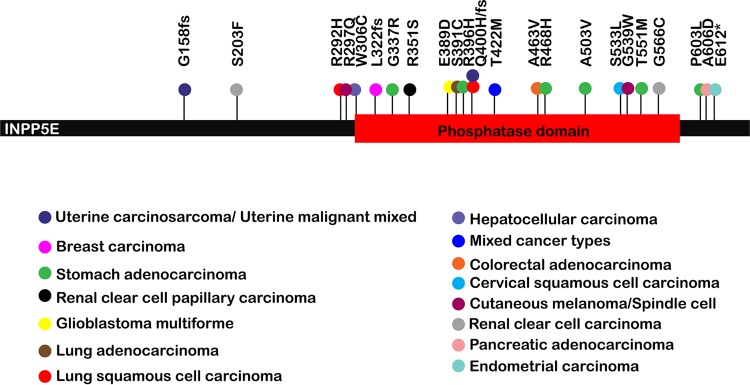
Acquired *INPP5E* mutations in cancer. Note that most cancer-associated mutations cluster within the INPP5E phosphatase domain. Cancer types are indicated by colors of mutation-associated circles as shown. The results shown here are based upon data generated by the TCGA Research Network (http://cancergenome.nih.gov/).

Chromosome instability is a hallmark of carcinogenesis (32). Somatic *INPP5E* abnormalities occur in cancer, although it is not clear whether loss of INPP5E promotes malignant transformation, as microarray studies reported both elevated and decreased *INPP5E* transcription in different malignancies (reviewed in references 14 and 50). Increased *INPP5E* transcription was reported in cervical cancers, uterine leiomyomas, and lymphomas (51–53), while decreased *INPP5E* transcription was found in gastric carcinomas and metastatic adenocarcinomas (54, 55). Since these microarray-based studies examined *INPP5E* expression but did not address the functionality of *INPP5E* transcripts, we independently analyzed somatic cancer-associated *INPP5E* mutations in the Cancer Genome Atlas (TCGA) database. We found that most cancer-associated *INPP5E* mutations cluster within the phosphatase domain (Fig. 13), suggesting that enzymatic activity may contribute to this phosphatase's tumor suppressor function. It is unknown whether germ line *INPP5E* mutations predispose to cancer: *INPP5E* mutations occur in only approximately 3% of Joubert syndrome (JBTS) patients (16), and many patients affected with this rare disorder die young. However, benign tongue tumors occur in JBTS (16, 56), and Burkitt lymphoma has been reported in Joubert syndrome (57). Since *Inpp5e* deficiency causes profound perinatal lethality in mice (17), future animal studies should employ inducible or tissue-specific knockout technology to explore the proposed tumor suppressor function of this phosphatase *in vivo*.

We conclude that INPP5E belongs to the expanding family of disease-associated phosphoinositide phosphatases that proofread mitosis to prevent aneuploidy. The tumor suppressor PTEN ensures chromosome integrity (58) and is phosphorylated by PLK1 (59). The oculocerebrorenal syndrome of Lowe (OCRL) phosphatase is recruited to the midbody by the Rab35-GTPase to regulate midbody-associated PI(4,5)P_2_ and reorganizes the actin cytoskeleton for abscission during cytokinesis (46). The PIPP/INPP5J phosphatase was shown to suppress breast cancer initiation and progression through negative regulation of oncogenic PI3K/AKT signaling (60). Our findings demonstrate new mitotic functions of INPP5E, thereby uncovering a new avenue through which INPP5E and phosphoinositides may direct human development and the prevention of cancer.

## MATERIALS AND METHODS

### Cell culture.

Primary human fibroblasts (HDFa) were purchased from ATCC. HeLa and HCT cells were a gift from D. Wade Clapp (Indiana University [IU]). All cells were cultured in Dulbecco modified Eagle medium (DMEM) with 10% fetal bovine serum (FBS) and 1% penicillin-streptomycin. *INPP5E* knockout HAP1 cells were purchased from Horizon Discovery (HZGHC002902c010) and maintained in Iscove modified Dulbecco medium (IMDM) with 10% FBS and 1% penicillin-streptomycin. *Inpp5e*^*flox/flox*^ MEFs were generated by crossing *Inpp5e*^*flox/+*^ mice, a gift from Stéphane Schurmans (University of Liege). MEFs were maintained in DMEM with 15% FBS, 1% l-glutamine, 1% sodium pyruvate, and 1% penicillin-streptomycin. All siRNAs were purchased from Ambion. Using siPORT NeoFX transfection reagent (Ambion), cells were reverse transfected in 6-well plates (37,440 cells per well) with 25 nM siRNA on day 1, then forward transfected with 25 nM siRNA on day 2, and then allowed 48 h of growth before harvesting or further processing.

To generate stable shRNA-expressing HeLa and fibroblast cells, lentiviral shRNA plasmids targeting luciferase (negative control; pLKO.1-puro luciferase shRNA control plasmid DNA [Sigma]) or *INPP5E* (pLKO.1-puro *INPP5E* shRNA TRCN0000082677 [Sigma]) were used to produce lentiviral particles as described previously (61). Upon transduction, cells were selected with 1 μg/ml puromycin.

INPP5E-depleted MEFs were generated by transduction of *Inpp5e*^*flox/flox*^ MEFs with lentivirus encoding green fluorescent protein (GFP)-fused Cre recombinase. Control cells were generated by transducing *Inpp5e*^*flox/flox*^ MEFs with lentivirus encoding GFP only. Transduced cells were sorted by GFP positivity via flow cytometry (Flow Cytometry Facility, IU Simon Cancer Center) before further processing.

For overexpression experiments, HeLa cells were transfected with 1 μg of the indicated plasmids using Lipofectamine LTX (Life Technologies) or X-tremeGENE HP (Roche). Cells were processed at 24 to 48 h posttransfection. GFP-INPP5E and DDK-INPP5E constructs were purchased from Origene (RG206984 and RC206984, respectively). The RFP–PLCδ-PH construct was a gift from Yang Sun (IU).

### Quantitative Western blotting.

Whole-cell lysates were prepared for Western blotting by incubating cells in M-PER mammalian protein extraction reagent (Life Technologies) with protease (Complete Mini, EDTA-free; Roche) and phosphatase inhibitors (Pierce Phosphatase Inhibitor Mini tablets; Thermo Scientific) on ice (10 min). Lysates were centrifuged at top speed in a microcentrifuge for 10 min. Prior to loading onto gels, samples were diluted with NuPAGE sample-reducing agent and NuPAGE lithium dodecyl sulfate (LDS) sample buffer (Life Technologies) and boiled (95°C, 5 min). Following protein separation on NuPAGE 4 to 12% polyacrylamide–bis-Tris gels (Life Technologies) and transfer to nitrocellulose, membranes were probed using the indicated primary antibodies. Fluorescent dye-conjugated secondary antibodies (Li-Cor Biosciences) were used for infrared fluorescence-based detection (Odyssey CLX). Protein levels were quantified by measuring the relative fluorescence intensities of bands (normalized against an actin or GAPDH [glyceraldehyde-3-phosphate dehydrogenase] loading control) using Image Studio 2.1 software.

### Deconvolution and SR microscopy.

Cells grown on ultrafine glass coverslips were fixed with 4% paraformaldehyde–PBS (Electron Microscopy Sciences) for 10 min at room temperature (RT). When indicated, soluble proteins were extracted prior to fixation with 0.1 to 0.2% Triton X-100–PBS (1 min at RT). Cells were next permeabilized and blocked in 0.2% Triton X-100–5% bovine serum albumin–PBS (1 h at RT) and then incubated with primary antibodies in PBS (1 h at RT or overnight at 4°C). Cells were washed with PBS (3 times for 5 min each at RT), incubated with fluorophore-conjugated secondary antibodies (Life Technologies) in PBS (1 h at RT), and washed with PBS as before. DNA was detected by counterstaining cells with Hoechst 33342 (Invitrogen). To detect actin, cells were stained with fluorophore-conjugated phalloidin (Life Technologies). Coverslips were mounted in SlowFade antifade reagent A (Life Technologies) and sealed with nail polish.

For deconvolution microscopy, images were acquired as a series of z-sections using a DeltaVision personalDx microscope (Applied Precision) fitted with 60× and 100× lenses and a charge-coupled device (CCD) camera, and deconvolved using SoftWoRx (10 iterations, conservative) (4). SR-SIM images were acquired with a Zeiss ELYRA PS.1 superresolution microscope using a 63× objective and the smallest z-section thickness possible. Laser powers and exposure time were kept consistent for all images in a given experiment. Images were processed in manual mode to preserve the raw intensity scale, with all other settings as for automatic processing, using a structured illumination (SIM) algorithm in Zen 2011 software. Postprocessing fluorescent-channel alignment was performed using slide-mounted TetraSpeck Microspheres (Life Technologies). All images were processed with Imaris (Bitplane). Images shown in figures represent individual z-sections of deconvolution or SIM stacks unless stated otherwise.

### Primary antibodies.

The primary antibodies used were as follows: rabbit anti-INPP5E (17797-1-AP; Proteintech); rabbit anti-INPP5E (HPA065758; Sigma), rabbit anti-DDK (14793S; Cell Signaling); rabbit antipericentrin (ab4448; Abcam); mouse anti-PLK1 (ab17056; Abcam); mouse anti-gamma-tubulin (GTU-88; Sigma); mouse phospho-Aurora (2914S; Cell Signaling); mouse anti-alpha-tubulin (A11126; Life Technologies); mouse anti-PI(4,5)P_2_ (Z-P045; Echelon Biosciences), rabbit anti-CENPA (2186S; Cell Signaling); mouse antiactin (A5441; Sigma); mouse anti-GAPDH (sc-365062; Santa Cruz Biotechnology), anti-phospho-H3 (9701L; Cell Signaling), and anti-cyclin B1 (4135S; Cell Signaling); and mouse anti-lamin A+C (ab40567; Abcam).

### Live cell imaging.

Cells were grown in DMEM plus 10% FBS plus 1% penicillin-streptomycin on Hi-Q4 four-chambered imaging plates (IBIDI) in the environmentally controlled chamber (5% CO_2_, 37°C) of a BioStation IM-Q (Nikon). Using the integrated microscope, phase-contrast and/or fluorescence time-lapse images were acquired every 2 min and analyzed using Imaris (Bitplane).

For live imaging of drug-treated HeLa cells, paclitaxel was added to the growth medium immediately before imaging (final concentration, 200 nM). MEFs were treated with 2 μM paclitaxel. For paclitaxel-PI(4,5)P_2_ treatment, PI(4,5)P_2_ [metabolically stable PI(4,5)P_2_ P-F4516; Echelon Biosciences] was combined with unlabeled Shuttle PIP Carrier 3 (P-9C3; Echelon Biosciences) per the manufacturer's instructions, and the complex was diluted to a final concentration of 10 μM in growth medium. Cells were grown in the PI(4,5)P_2_-carrier complex-containing medium for 44 h, at which point paclitaxel was added (final concentration, 200 nM) and time-lapse imaging was initiated.

### Inhibition of PI(4,5)P_2_ synthesis.

HeLa cells stably expressing sh*INPP5E* were treated with 100 nM UNC3230 (Tocris) or dimethyl sulfoxide (DMSO) for 24 h. Paclitaxel was added to the growth medium at a final concentration of 200 nM. At 22 h after paclitaxel exposure, cells were observed via live time-lapse imaging or fixed for immunofluorescence.

### Quantification of PI(4,5)P_2_.

Phosphoinositides were extracted from cells using the NeoBead PIP purification system from Echelon Bioscience (P-B999). PI(4,5)P_2_ was then quantified via PI(4,5)P_2_ Mass ELISA (Echelon Biosciences; K-4500). Both procedures were performed per the manufacturer's instructions.

### Micronucleation assay.

Primary human fibroblasts stably expressing shRNAs targeting luciferase or *INPP5E* were plated on coverslips, treated with 2 μg/ml cytochalasin B in growth medium for 24 h, fixed, and immunostained for CENPA as described above. Cells were counterstained with fluorophore-conjugated phalloidin and Hoechst stain for detection of cell borders and DNA, respectively. Images were acquired and analyzed via deconvolution microscopy (described above). Cells were scored for the presence of micronuclei, which were defined as kinetochore positive or negative based on the respective presence or absence of CENPA signal within the stack of z-images spanning the entire volume of micronuclei.

### Microtubule cold-destabilization assay.

At 48 h posttransfection with the indicated siRNAs, coverslips with live HeLa cells were transferred to individual wells on 12-well plates, submerged in cold DMEM plus 10% FBS plus 1% penicillin-streptomycin, and incubated at 4°C (1 h). The medium was then aspirated, replaced with medium prewarmed to 37°C, and left for 15 s. Cells were then immediately fixed and processed for immunofluorescence as described above.

For PIP_2_ treatment, PI(4,5)P_2_ [metabolically stable PI(4,5)P_2_ P-F4516; Echelon Biosciences] was combined with unlabeled Shuttle PIP Carrier 3 (P-9C3; Echelon Biosciences) in accordance with the manufacturer's instructions, and the complex was diluted to a final concentration of 20 μM in growth medium. Cells were grown in the PI(4,5)P_2_-carrier complex-containing medium for 24 h before the microtubule cold-destabilization assay was performed as described above.

### Cell cycle assays.

HCT cells were arrested in G_1_ by starvation (48 h) and released by reintroduction of 10% FBS as described previously (54). Cells were harvested at the indicated time points for Western blotting as described above or for flow cytometry as follows. Cells were detached using HyQTase cell detachment reagent (HyClone), washed twice with cold PBS, fixed in cold 70% ethanol overnight (−20°C), washed with PBS twice, and then stained with FxCycle PI/RNase staining solution (Life Technologies). Cell cycle profiles were generated using a FACSCalibur machine (BD) and analyzed with ModFit LT software (Verity Software House). For G_2_/mitotic release, HeLa cells were G_2_ arrested with 9 μM RO3306 (EMD Millipore) for 24 h (62) and released by four 5-min washes with drug-free medium at 37°C.

### Metaphase chromosome spreads.

Human fibroblasts were arrested in metaphase with 1 μg/ml colcemid (2 h, 37°C) (KaryoMAX colcemid; Life Technologies), trypsinized, resuspended in serum-containing medium, centrifuged (1,500 rpm, 5 min), resuspended in 5 ml of 75 mM KCl, and incubated at RT (10 min). Seven drops of fresh 3:1 methanol-acetic acid fixative were then added, and cells were centrifuged (1,500 rpm, 5 min). After removing all but 100 μl of the fixative, cells were resuspended in 4.5 ml of fresh fixative added dropwise while vortexing at low speed and then incubated in fixative overnight (4°C), centrifuged (1,500 rpm, 5 min), resuspended in 1 ml of fresh fixative, pelleted in a microcentrifuge at top speed, washed with fixative, again pelleted in a microcentrifuge at top speed for 2 min, and resuspended in 250 μl fixative. One hundred microliters of suspension was dropped onto ethanol-precleaned microscope slides and dried in a fume hood (1 h). The slides were washed in ultrapure water and mounted in DAPI (4′,6′-diamidino-2-phenylindole)-containing Vectashield medium (Vector Laboratories) prior to deconvolution microscopy.

### Statistics.

All statistical analyses were performed using GraphPad Prism. All graphs show mean values ± standard errors of the mean (SEM) unless indicated otherwise.
